# Phosphorylation and Ubiquitylation Regulate Protein Trafficking, Signaling, and the Biogenesis of Primary Cilia

**DOI:** 10.3389/fcell.2021.664279

**Published:** 2021-04-12

**Authors:** Elena A. May, Tommy J. Sroka, David U. Mick

**Affiliations:** ^1^Center of Human and Molecular Biology (ZHMB), Saarland University School of Medicine, Homburg, Germany; ^2^Center for Molecular Signaling (PZMS), Department of Medical Biochemistry and Molecular Biology, Saarland University School of Medicine, Homburg, Germany

**Keywords:** primary cilia, post-translational modification, cell signaling, ciliogenesis, Hedgehog signaling, phosphorylation, ubiquitylation

## Abstract

The primary cilium is a solitary, microtubule-based membrane protrusion extending from the surface of quiescent cells that senses the cellular environment and triggers specific cellular responses. The functions of primary cilia require not only numerous different components but also their regulated interplay. The cilium performs highly dynamic processes, such as cell cycle-dependent assembly and disassembly as well as delivery, modification, and removal of signaling components to perceive and process external signals. On a molecular level, these processes often rely on a stringent control of key modulatory proteins, of which the activity, localization, and stability are regulated by post-translational modifications (PTMs). While an increasing number of PTMs on ciliary components are being revealed, our knowledge on the identity of the modifying enzymes and their modulation is still limited. Here, we highlight recent findings on cilia-specific phosphorylation and ubiquitylation events. Shedding new light onto the molecular mechanisms that regulate the sensitive equilibrium required to maintain and remodel primary cilia functions, we discuss their implications for cilia biogenesis, protein trafficking, and cilia signaling processes.

## Introduction

**FIGURE 1:**
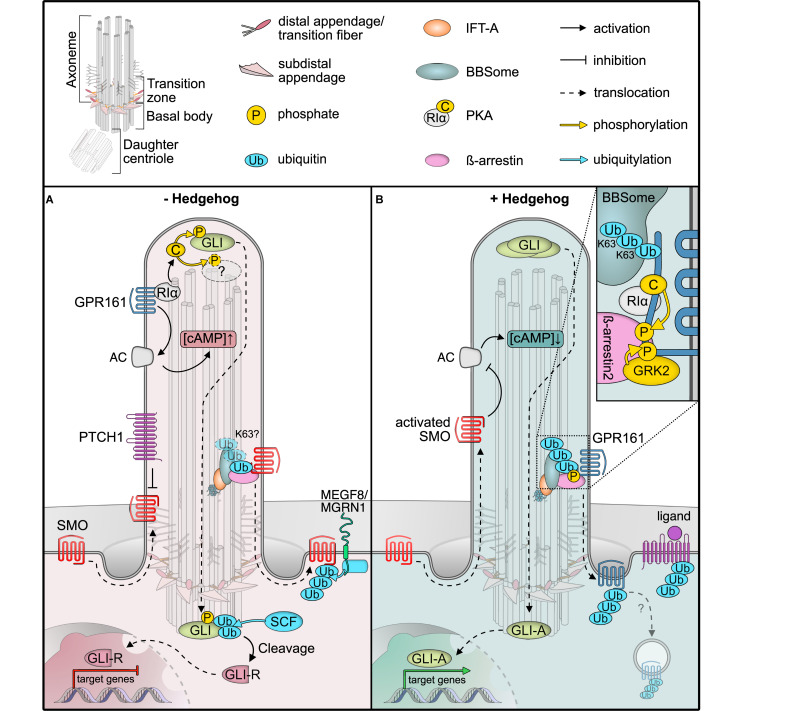
Post-translational modifications (PTMs) regulating Hedgehog signaling. **(A)** In unstimulated cells, the GPCR SMO constantly surveys the cilium without accumulation, due to constant removal in a ubiquitin (Ub) and BBSome-dependent manner. The Hedgehog receptor PTCH1 suppresses SMO, and the constitutively active GPCR GPR161 is retained in cilia. GPR161 stimulates adenylyl cyclases (AC) to generate cAMP. High cAMP is sensed by the regulatory PKA subunit Iα (RIα), which releases the PKA catalytic subunit (C) to phosphorylate target proteins, such as GLI transcription factors. GLI phosphorylation leads to ubiquitylation and proteolytic cleavage to GLI repressor forms (GLI-R) that repress target gene expression in the nucleus. The ubiquitin ligase MEGF is recruited to the plasma membrane by MGRN1 where it ubiquitinylates SMO for subsequent degradation. **(B)** In the presence of Hedgehog ligands, PTCH1 exits the primary cilium, presumably in a Ub-dependent fashion, leading to SMO activation and accumulation. GPR161 in turn is phosphorylated by GRK2 (and PKA). GPR161 phosphorylation is sensed by β-arrestin2, which leads to ubiquitylation and BBSome-mediated removal of GPR161 together with PKA from the primary cilium. Together with a drop in cAMP levels, GLIs are no longer phosphorylated and full-length GLIs activate target genes in the nucleus. After removal from cilia, the mechanism by which GPR161 is internalized remains unclear.

Primary cilia are dynamic cellular signaling compartments of the plasma membrane (Garcia et al., 2018; Anvarian et al., 2019) composed of a membrane-surrounded microtubule core, termed the axoneme. The axoneme emerges from a matured mother centriole, the so-called basal body, that connects to the plasma membrane via distinct appendages, the transition fibers (see Figure 1). Primary cilia are indispensable for embryonic development and cell differentiation. Consequently, defective primary cilia give rise to severe human diseases, known as ciliopathies, that are commonly caused by aberrant ciliary signaling processes (Baker and Beales, 2009; Reiter and Leroux, 2017). On a molecular level, observed defects comprise not only signaling components but also the protein machinery that is required to build and maintain cilia (Sánchez and Dynlacht, 2016; Breslow and Holland, 2019). Therefore, ciliopathy genes also include protein trafficking components, such as the cilia-specific intraflagellar transport (IFT) complexes, IFT-A and IFT-B (Webb et al., 2020), and all eight subunits of the BBSome, defects of which cause Bardet–Biedl Syndrome (Jin et al., 2010; Forsythe et al., 2018). IFT complexes transport cargoes along the axoneme in an anterograde and retrograde fashion with the help of specific kinesin and dynein motors, respectively (Satir and Christensen, 2007). The ciliary membrane does not fully enclose the ciliary compartment at the proximal end, where it is separated from the cytosol by the transition zone (Yang et al., 2015). A concerted interplay of IFT complexes, the BBSome, transition fibers, and the transition zone enables select proteins to enter or exit the cilium (Garcia-Gonzalo and Reiter, 2017; Gonçalves and Pelletier, 2017).

Post-translational modification (PTM) is a fundamental principle in molecular biology referring to the modulation of protein properties by covalent attachment of small molecules. PTMs are catalyzed by various antagonistic enzymatic activities that modify target proteins at specific locations (Vu et al., 2018). For instance, phosphorylation can modulate interaction surfaces or lead to intramolecular rearrangements that alter enzymatic activities. Protein kinases phosphorylate their substrates at specific consensus sites consisting of only a few amino acids. Moreover, they are often targets of phosphorylation themselves, which results in phosphorylation cascades that are typically found in cellular signaling processes (Miller and Turk, 2018). Opposingly, protein phosphatases act on hundreds of different substrates to revert phosphorylations (Bertolotti, 2018). Compared to phosphorylation, ubiquitylation requires a more elaborate machinery. Ubiquitin is a small, 8.5-kDa protein that is usually attached to lysine residues of target proteins (Swatek and Komander, 2016; Yau and Rape, 2016). The enzymatic cascade of ubiquitylation involves E1 activating, E2 conjugating, and E3 ligating enzymes. While the E1 and E2 enzymes supply reactive ubiquitin molecules, the vast number of different E3 ubiquitin ligases determines substrate specificity. Similarly, deubiquitylating enzymes (DUBs) are highly specific with only a few substrates per enzyme (Clague et al., 2019). Ubiquitin contains seven lysine residues, to which further ubiquitin molecules can be added to generate poly-ubiquitin chains. Depending on the lysine residue, ubiquitin chains are differentiated into several linkage types that have been implicated in specific functions. K48- and K29-linked ubiquitins, for example, are the main linkage types associated with proteasomal degradation of target proteins, while the K63 chains and mono-ubiquitin are often times involved in protein trafficking events (Swatek and Komander, 2016).

The dynamic nature of PTMs is critical for most cellular processes and is extensively studied in protein trafficking and cell signaling (Patwardhan et al., 2021). The central role of the primary cilium as a cellular signaling hub suggests that PTMs regulate core ciliary functions. In addition to phosphorylation and ubiquitylation, ciliary proteins are targets of diverse modifications, such as acetylation (Kerek et al., 2021), SUMOylation (McIntyre et al., 2015), and methylation (Yeyati et al., 2017). Several lipid modifications (including acylation, myristoylation, palmitoylation, and prenylation) of ciliary proteins have also been involved in protein trafficking, membrane tethering, and protein stability (Roy and Marin, 2019). Moreover, ciliary microtubules are extensively acetylated, detyrosinated, glutamylated, and glycylated, which reflects axoneme maturation and affects axoneme assembly, protein interaction, and stability (Janke and Magiera, 2020). In the following, we focus on phosphorylation and ubiquitylation and discuss recent findings on their involvement in regulating cilia formation and signaling.

## Ciliary Signaling

Conceptually, primary cilia are believed to function as cell type-specific micro-compartments with diverse compositions including receptors to receive, mediators to process, and effectors to transmit signals to the rest of the cell (Sung and Leroux, 2013; Nachury and Mick, 2019). Despite a large variety of receptors, far fewer mediators are commonly used in cellular signaling processes. Cyclic nucleotides or calcium ions are second messengers, the concentrations of which are interpreted by specific enzymes to further transmit signals via PTMs (Hilgendorf et al., 2019; Sherpa et al., 2019; Tajhya and Delling, 2020). To communicate with the rest of the cell, effectors are transported into and out of cilia in a dynamic fashion, which allows their modification according to the signaling status (Niewiadomski et al., 2019). This general principle highlights the tight connection between cilia signaling and protein trafficking. Apart from the IFT complexes, cilia require a multitude of additional factors to convey ciliary signals, which involves not only common protein trafficking components, such as β-arrestins, but also cilia-specific machinery, including the BBSome or the Tubby family of proteins (Mukhopadhyay and Jackson, 2011). While the inventory of primary cilia continues to expand (Mick et al., 2015; Kohli et al., 2017; May et al., 2021), the number of enzymes, which catalyze PTMs and have been unambiguously shown to localize to primary cilia, is limited. Nonetheless, we are beginning to unravel how ciliary signaling dynamics can be established as we identify more and more targets of PTMs in cilia.

### Hedgehog Signaling

One hallmark ciliary signaling pathway that highlights the dynamics in PTMs is Hedgehog signaling in vertebrates (Figure 1; Gigante and Caspary, 2020). An elegantly orchestrated interplay of positive and negative regulators in Hedgehog signaling allows for the correct patterning of the developing embryo, in addition to maintaining adult tissue homeostasis (Shimada et al., 2019). Gradients of the hedgehog morphogens ultimately result in finely tuned levels of active GLI transcription factors that determine target gene expression (Briscoe and Novitch, 2008). In the absence of Hedgehog morphogens, their receptor Patched (PTCH1) localizes to the primary cilium (Rohatgi et al., 2007), while the key Hedgehog effector and G protein-coupled receptor (GPCR) Smoothened (SMO) surveys the primary cilium by shuttling in and out without appreciable local accumulation (Figure 1A; Kim et al., 2009; Goetz and Anderson, 2010). A second GPCR, the constitutively active GPR161, stimulates ciliary adenylyl cyclases to increase cAMP levels within cilia and thereby activates the cAMP-dependent protein kinase (PKA) (Mukhopadhyay et al., 2013). GPR161 also fulfills a second function in PKA signaling, as it serves as an atypical A-kinase anchoring protein (AKAP) that targets PKA to cilia (Bachmann et al., 2016). Here, it tethers to the cilia-resident PKA regulatory subunit RIα that senses ciliary cAMP (Mick et al., 2015). At high ciliary cAMP levels in unstimulated cells, PKA-RIα binds cAMP and releases the catalytic PKA-C subunit (Figure 1A; Taylor et al., 2004). Free PKA-C can phosphorylate and regulate target proteins, such as the GLI transcription factors that convey the signaling status to the nucleus (Tuson et al., 2011; Niewiadomski et al., 2014). PKA-mediated phosphorylation of GLIs is a pre-requisite for their proteolytical cleavage to yield repressor forms that block the transcription of target genes (Figure 1A). GLI transcription factors are precisely regulated by a variety of activating and deactivating PTMs, which include activating phosphorylations at the N-terminal repressor domain, and two clusters of PKA phosphorylation sites on the activator domain. PKA phosphorylation precedes further phosphorylation by CK1 and GSK3β, which in turn recruits the SCF E3 ubiquitin ligase that marks GLIs for proteolytic processing by the proteasome (Kong et al., 2019; Niewiadomski et al., 2019).

Upon Hedgehog ligand binding, PTCH1 exits the primary cilium and SMO is activated and retained in cilia, whereas GPR161 is removed (Rohatgi et al., 2007; Gigante and Caspary, 2020). As the adenylyl cyclase inhibitory SMO replaces the stimulating GPR161, ciliary cAMP decreases (Mukhopadhyay et al., 2013). Consequently, PKA activity ceases and the GLI transcription factors are no longer phosphorylated and further processed, such that they can function as activators to initiate target gene expression in the nucleus (Figure 1B).

One central element in Hedgehog signaling is the dynamic re-localization of the components involved. Similar to other cellular protein trafficking mechanisms, PTMs control the localization of Hedgehog signaling proteins, which is particularly well-studied for GPR161 (Mukhopadhyay et al., 2013; Pal et al., 2016). GPR161’s C-terminal tail not only contains the AKAP binding domain for PKA but also several protein kinase consensus sites, including one for PKA (Bachmann et al., 2016). Upon Hedgehog pathway activation, the C-terminal tail of GPR161 is phosphorylated by GRK2 and presumably PKA (Bachmann et al., 2016; Pal et al., 2016; May et al., 2021). GRK-mediated phosphorylation recruits the molecular sensor of activated GPCRs, specifically β-arrestin2, which is required for the removal of activated GPCRs from cilia (Figure 1B; Pal et al., 2016). Consequently, GPR161 exits cilia together with its binding partner PKA (May et al., 2021). Thereby, PKA activity in cilia is inhibited by two mechanisms: (i) reducing cAMP levels and (ii) removing PKA itself.

As exemplified by the GLI transcription factors and GPR161, specific phosphorylations are often catalyzed by individual kinases; however, our knowledge of specific protein phosphatases that antagonize these phosphorylations in cilia is still rudimentary. The protein phosphatases PP1 and PP2A have been reported to dephosphorylate SMO to dampen Hh signaling in *Drosophila* (Su et al., 2011; Liu et al., 2020). However, since primary cilia are dispensable for *Drosophila* Hh signal transduction, it remains unclear whether PP1 and PP2A also function within primary cilia. Mass spectrometric analyses have identified PP1 subunits in isolated *Chlamydomonas* cilia (Pazour et al., 2005) and PP2A subunits in primary cilia of kidney epithelial cells (Ishikawa et al., 2012), but these findings still await confirmation by independent methods. In contrast, lipid phosphatases, such as the inositol polyphosphate-5-phosphatase E, have been unambiguously shown to localize to primary cilia, where they modulate ciliary signal transduction by regulating protein trafficking (Chávez et al., 2015; Garcia-Gonzalo et al., 2015). Ciliary lipid phosphatase activities create a specific phosphatidylinositide phosphate environment that is required for efficient ciliary signaling.

A recent study investigated the involvement of ubiquitin in Hedgehog signaling by fusing mono-ubiquitin to the C-terminus of SMO (Desai et al., 2020). The SMO-Ub fusion accumulated in cilia in the absence of stimulation in IFT and BBSome mutants but failed to accumulate in cilia after Hedgehog pathway activation in wild type cells (Desai et al., 2020). These findings indicate that ubiquitin is required for the removal of SMO from cilia by a process involving IFT and the BBSome. Moreover, β-arrestin2 was shown to mediate the ubiquitylation of GPR161 in response to Hedgehog pathway activation (Shinde et al., 2020), before GPR161 exits the primary cilium in a BBSome-dependent fashion (Ye et al., 2018). More evidence for the central role of ubiquitylation for cilia trafficking comes from mutational analysis of the Hedgehog receptor PTCH1. PTCH1 harbors two E3 ubiquitin ligase recognition motifs and remains in the cilium when both motifs are mutated, even upon stimulation with Hedgehog ligands (Kim J.C. et al., 2015). SMO has been reported to be a target of the ubiquitin ligase HERC4 (Jiang et al., 2019). Furthermore, ubiquitylation of SMO by a complex of the E3 ubiquitin ligase MGRN1 and the plasma membrane protein MEGF8 serves as a signal for proteasomal degradation (Kong et al., 2020). Yet, whether these ubiquitin ligases are directly involved in the IFT-dependent retrieval of SMO awaits experimental validation.

Molecular dissection of ubiquitylation may help to decipher the different functions of ubiquitin in regulating ciliary proteins. Upon Hedgehog pathway activation, specifically K63-linked ubiquitin chains increase in primary cilia upon GPCR activation or in BBSome mutants (Shinde et al., 2020). This suggests that K63 ubiquitin chains function as export signals for ciliary proteins, which are recognized by the BBSome (Desai et al., 2020; Shinde et al., 2020). In BBSome mutant mice, photoreceptor outer segments, which are uniquely modified cilia that harbor the entire signaling cascade for visual phototransduction, accumulate more than 100 proteins that are absent in wild types (Datta et al., 2015). Based on these findings, the BBSome has been proposed to mediate the removal of unwanted proteins from cilia and, therefore, may function as an important mediator of a ciliary protein quality control network (Shinde et al., 2020). Additional components, such as the AAA-ATPase VCP or the ubiquitin-regulatory X domain protein UBXN10, have been shown to localize to primary cilia (Mick et al., 2015; Raman et al., 2015). While data from trypanosomes indicate that the BBSome may directly recognize ubiquitin as it can be enriched on ubiquitin-agarose resin (Langousis et al., 2016), it does not contain canonical ubiquitin binding domains. How ubiquitylated proteins are recognized in cilia on a molecular level and what enzymatic activities regulate ubiquitylation within cilia remains to be established. The E3 ubiquitin ligase CBL is recruited to cilia in response to PDGFRα signaling (Schmid et al., 2018) and the deubiquitylase UBPY/USP8 has been reported to antagonize SMO ubiquitylation in *Drosophila* (Ma et al., 2016). These findings seem promising starting points for future studies elucidating the cilia-specific ubiquitylation network.

## Cilium Dynamics

### Cilium Assembly—A Primary Cilium Is (Re)born

**FIGURE 2:**
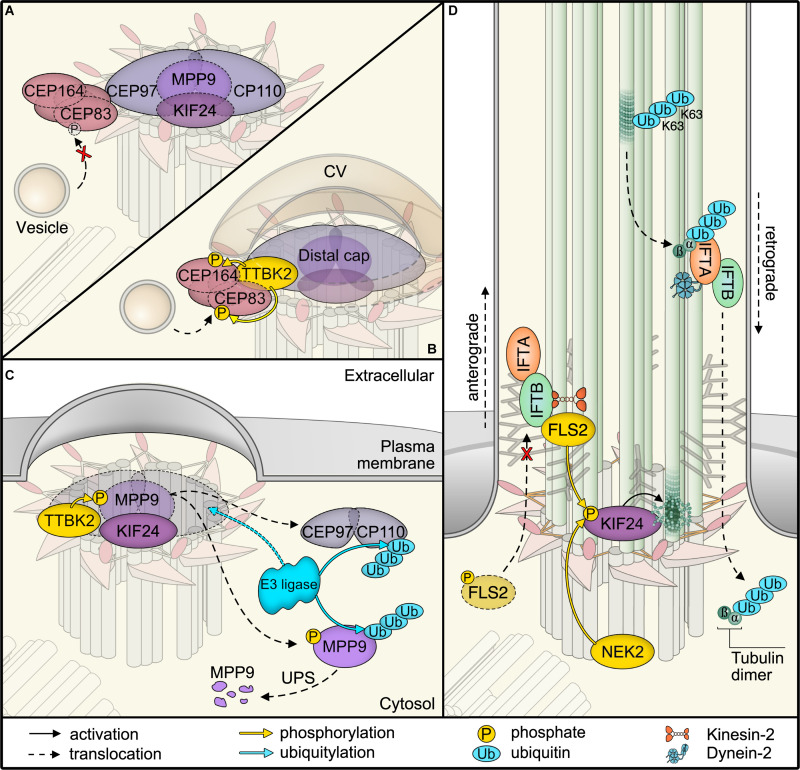
Post-translational modifications (PTMs) in primary cilia assembly and disassembly. **(A)** Mother centriolar distal appendage components CEP164 and CEP83 are shown in red. The distal end proteins KIF24 and MPP9 recruit the capping protein complex CEP97–CP110 to block axoneme extension. Note that only one cap is shown for simplicity, while each microtubule triplet is capped by one complex. Unphosphorylated CEP83 diminishes ciliary vesicle recruitment **(B)** CEP164 recruits the kinase TTBK2 that phosphorylates CEP164 and CEP83. Recruitment and activity of TTBK2 enables subsequent steps of cilia assembly such as formation of the ciliary vesicle (CV). **(C)** TTBK2 phosphorylates MPP9, which results in ubiquitylation and dissociation of MPP9 and the remaining CEP97–CP110 complex from the distal centriolar end. Several E3 ubiquitin ligase complexes have been implicated in modifying distal cap components, while the precise location of ubiquitylation has not been determined (see text for details). Ultimately, MPP9, CEP97, and CP110 are degraded by the proteasome (UPS) and ciliary growth can be initiated. **(D)** Diagram of fully assembled primary cilium. The microtubule depolymerizing kinesin KIF24 has been implicated in microtubule disassembly. Phosphorylation of KIF24 by NEK2 stimulates KIF24 activity. In *Chlamydomonas*, dephosphorylated FLS2 enters cilia by binding to IFT-B and phosphorylates the KIF24 homolog. Upon disassembly, tubulins are ubiquitylated by unknown mechanisms. IFT-A binds to K63-linked ubiquitin chains and mediates removal.

Ciliogenesis, i.e., the formation of cilia, is another dynamic process that is regulated by specific phosphorylation and ubiquitylation events (Cao et al., 2009; Shearer and Saunders, 2016). A specialized maternal centriole, the so-called basal body, templates the cilium. Yet, mother and daughter centrioles also form centrosomes required for spindle apparatus formation and chromosome segregation in metaphase. These two alternative roles of the mother centriole necessitate a cell cycle-dependent assembly and disassembly of primary cilia (Wang and Dynlacht, 2018; Breslow and Holland, 2019). Depending on cell type, the mother centriole takes one of two different routes to form a cilium, starting either directly at the plasma membrane (termed extracellular pathway) or within the cell (Bernabé-Rubio and Alonso, 2017; Kumar and Reiter, 2021). Here, we will be focusing on the intracellular pathway, which occurs in several steps (see Figure 2): (i) maturation of the mother centriole and acquisition of so-called distal and subdistal appendages, (ii) recruitment of a growing ciliary vesicle (the future ciliary membrane) to the mother centriole, (iii) separation of the ciliary compartment by the formation of the transition zone, (iv) extension of the ciliary axoneme, and (v) docking of the basal body and final fusion with the plasma membrane. While this process has been described on an ultrastructural level more than half a century ago (Sorokin, 1968), we are still discovering an increasing number of the required factors such as RABs and EHD family proteins that are involved in membrane recruitment (Lu et al., 2015; Blacque et al., 2018) and are just beginning to understand their regulation.

A central kinase that determines cilium formation is the Tau tubulin kinase 2 (TTBK2) (Tomizawa et al., 2001; Goetz et al., 2012). TTBK2 loss was originally reported to allow basal body docking to the plasma membrane, while blocking transition zone formation and ciliary shaft elongation. In actively proliferating cells, the distal ends of both mother and daughter centrioles are capped by protein complexes of CP110 and CEP97 that suppress cilia formation (Spektor et al., 2007; Schmidt et al., 2009). Recruitment of these caps seems to follow a hierarchical scheme, the precise order of which awaits clarification (Ye et al., 2014; Tsai et al., 2019). One central component involved in ciliogenesis is the microtubule-depolymerizing kinesin KIF24 (Kobayashi et al., 2011). KIF24 recruits the M-Phase phosphoprotein MPP9, which is required for the assembly of CEP97–CP110 complexes at the distal ends of centrioles (Figure 2A; Huang et al., 2018). The specific removal of the CEP97–CP110 complex relies on TTBK2 phosphorylation (Figures 2B,C; Goetz et al., 2012; Čajánek and Nigg, 2014; Huang et al., 2018). To ensure specificity of distal end uncapping and thereby cilium formation at the mother centriole, it is the distal appendage protein CEP164 that recruits TTBK2 (Schmidt et al., 2012; Čajánek and Nigg, 2014). TTBK2 has recently been shown to phosphorylate distal appendage proteins, such as CEP164 and CEP83 (Bernatik et al., 2020), which is required for efficient vesicle recruitment (Figure 2B; Lo et al., 2019). Notably, TTBK2 phosphorylates MPP9 resulting in the loss of MPP9 and the CEP97–CP110 complex from the distal centriolar end (Figure 2C; Huang et al., 2018). Moreover, with the onset of cilia formation, MPP9 is ubiquitylated and degraded by the proteasome. Although the precise ubiquitin linkage type has not been determined yet, many molecular details of MPP9 PTM have been resolved. Intriguingly, one identified ubiquitylation site in MPP9 is flanked by two phosphorylation sites. Phosphorylation-deficient mutants show reduced ubiquitylation and consequently stabilize MPP9 (Huang et al., 2018). This finding highlights a typical PTM cascade and suggests that the phosphorylation status determines MPP9 stability. Similar to MPP9, the CEP97–CP110 complex is subject to proteasomal degradation when ciliation is initiated (Spektor et al., 2007; Nagai et al., 2018). CP110 has been shown to be a target of the SCF ubiquitin ligase complex and a substrate of the E3 ubiquitin ligase UBR5 in an *in vitro* ubiquitylation assay (D’Angiolella et al., 2010; Hossain et al., 2017). Additionally, CEP97 degradation is suppressed after knockdown of the CUL3 E3 ligase, and therefore, it remains bound to CP110 at centrioles and inhibits ciliogenesis (Nagai et al., 2018). UBR5 has been found at centrosomes and CUL3 has been suggested to localize specifically to mother centrioles (Moghe et al., 2012; Nagai et al., 2018), where it may ubiquitylate Aurora kinase A, a central regulator of the cell cycle and promoter of cilium disassembly (Pugacheva et al., 2007). It will be interesting to investigate whether these ubiquitin ligases converge on the same targets and whether ubiquitylation is the cause or consequence of CEP97–CP110 removal. Also, how precisely ubiquitylation can be regulated and what role DUBs, such as USP33 that targets CP110 (Li et al., 2013), are playing in ciliogenesis need to be addressed in future studies.

### Cilium Disassembly

In contrast to cilia formation, we are just beginning to understand the molecular details of how cilia are dismantled to allow cell cycle re-entry (Liang et al., 2016; Breslow and Holland, 2019). NEK2, a kinase predominantly expressed in the S and G2 phases of the cell cycle, has been proposed to promote cilium disassembly (Figure 2D; Kim S. et al., 2015). Among several targets, NEK2 phosphorylates and stimulates the microtubule-depolymerizing KIF24 at the distal centriolar ends (Kim S. et al., 2015). This may not only block unwanted cilium assembly but also shift the balance toward disassembly when resting cells re-enter the cell cycle (Kim S. et al., 2015; Viol et al., 2020). In support of a central role for KIF24 in cilium disassembly, a recent study identified FLS2 as a CDK-like kinase that phosphorylates the KIF24 ortholog CrKIF13 in *Chlamydomonas*, allowing efficient cilia disassembly (Figure 2D; Zhao et al., 2020). In turn, phosphorylated FLS2 showed lower activity and appears to be dephosphorylated upon cilia disassembly when it enters cilia by binding to the IFT-B component IFT70 (Zhao et al., 2020). While the precise mechanisms of regulation still need to be established, it is tempting to speculate that dephosphorylation of FLS2 may not only alter its kinase activity but also unmask targeting signals for IFT. This suggests a mechanism by which an active kinase can be directed into cilia to promote disassembly by phosphorylating specific targets such as the microtubule-depolymerizing kinesin KIF24.

In cilia of *Chlamydomonas*, a ubiquitin conjugation system has been identified more than a decade ago (Huang et al., 2009), yet the involvement of ubiquitin in cilia disassembly has only recently been demonstrated. Despite a massive rise in the ubiquitin levels in shortening cilia, semi-quantitative mass spectrometric analysis of a temperature-sensitive *Chlamydomonas* model has only detected an increase in ubiquitylation of α-tubulin and ubiquitin itself (Wang et al., 2019). The study further revealed α-tubulin poly-ubiquitylation by K63 chains, which allows binding to the IFT-A subunit IFT139 for tubulin removal via retrograde IFT (Figure 2D; Wang et al., 2019). Intriguingly, the authors also observed an increase in K11 and K48 chains in response to cilia shortening. K11 chains are also assembled by the anaphase-promoting complex to drive proteasomal degradation of substrates during mitosis, suggesting potential mechanisms for cell cycle-dependent regulation (Matsumoto et al., 2010).

## Outlook

As we are gathering increasing evidence for the existence of a ciliary ubiquitylation machinery involved in protein trafficking, signaling, disassembly, and potentially protein quality control, its identity remains elusive. Similarly, antagonistic cilia-specific DUBs as well as protein phosphatases that counterbalance known kinases await their identification. Powerful unbiased genetic and proteomic screening technologies have been applied to primary cilia (Mick et al., 2015; Kohli et al., 2017; Breslow et al., 2018; Pusapati et al., 2018) and promise to reveal the missing links that modulate manifold dynamic processes in cilia by PTM.

## Author Contributions

EM wrote the first draft of the manuscript. TS conceptualized and prepared the figures. DM wrote sections of the manuscript. All authors contributed to the conception of the article, manuscript revision, and read and approved the submitted version.

## Conflict of Interest

The authors declare that the research was conducted in the absence of any commercial or financial relationships that could be construed as a potential conflict of interest.
